# Conformational Selection of a Tryptophan Side Chain Drives the Generalized Increase in Activity of PET Hydrolases through a Ser/Ile Double Mutation

**DOI:** 10.1021/acsorginorgau.2c00054

**Published:** 2023-01-09

**Authors:** Alessandro Crnjar, Aransa Griñen, Shina C. L. Kamerlin, César A. Ramírez-Sarmiento

**Affiliations:** † Department of Chemistry—BMC, Uppsala University, BMC Box 576, S-751 23 Uppsala, Sweden; ‡ Institute for Biological and Medical Engineering, Schools of Engineering, Medicine and Biological Sciences, Pontificia Universidad Católica de Chile, Av. Vicuña Mackenna 4860, Santiago 7820436, Chile; § ANID—Millennium Science Initiative Program—Millennium Institute for Integrative Biology (iBio), Av. Libertador Bernardo O’Higgins 340, Santiago 8331150, Chile; ∥ School of Chemistry and Biochemistry, Georgia Institute of Technology, 901 Atlantic Drive NW, Atlanta, Georgia 30332-0400, United States

**Keywords:** PET hydrolases, plastic recycling, molecular dynamics, metadynamics, enzyme design

## Abstract

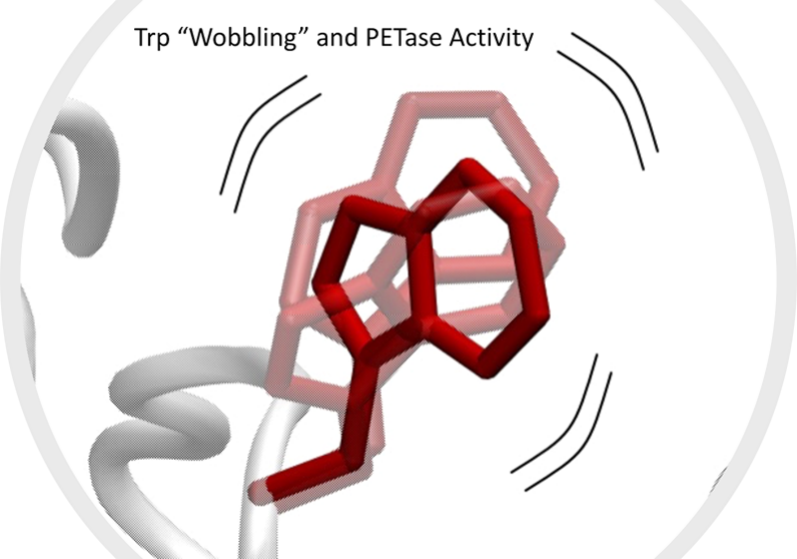

Poly(ethylene terephthalate) (PET) is the most common polyester plastic in the packaging industry and a major source of environmental pollution due to its single use. Several enzymes, termed PET hydrolases, have been found to hydrolyze this polymer at different temperatures, with the enzyme from *Ideonella sakaiensis* (*Is*PETase) having optimal catalytic activity at 30–35 °C. Crystal structures of *Is*PETase have revealed that the side chain of a conserved tryptophan residue within an active site loop (W185) shifts between three conformations to enable substrate binding and product release. This is facilitated by two residues unique to *Is*PETase, S214 and I218. When these residues are inserted into other PET hydrolases in place of the otherwise strictly conserved histidine and phenylalanine residues found at their respective positions, they enhance activity and decrease *T*_opt_. Herein, we combine molecular dynamics and well-tempered metadynamics simulations to investigate dynamic changes of the S214/I218 and H214/F218 variants of *Is*PETase, as well as three other mesophilic and thermophilic PET hydrolases, at their respective temperature and pH optima. Our simulations show that the S214/I218 insertion both increases the flexibility of active site loop regions harboring key catalytic residues and the conserved tryptophan and expands the conformational plasticity of this tryptophan side chain, enabling the conformational transitions that allow for substrate binding and product release in *Is*PETase. The observed catalytic enhancement caused by this substitution in other PET hydrolases appears to be due to conformational selection, by capturing the conformational ensemble observed in *Is*PETase.

## Introduction

The inexpensive manufacturing, long-term durability, and high resistance to degradation of plastics have led to its concerning accumulation as waste in landfills and oceans at rates that parallel its annual production.^1^ The primary sources of plastic waste are single-use or short-lifetime consumer plastics, mainly poly(ethylene terephthalate) (PET), polyethylene (PE), and polypropylene (PP), with PET being the predominant polymer in postconsumer domestic plastic waste collected in countries such as the UK (40% abundance).^2^

Although PET is recognized as one of the most recyclable plastics due to the vast presence of mechanical recycling industries, it can only endure a limited number of cycles before its properties are compromised. For example, the tensile and impact strength of virgin PET used in bottles decreases almost linearly within the first three extrusion cycles,^3^ whereas its ductility is reduced from >200 to <10% elongation at break upon mechanical recycling.^4^ Consequently, recycled PET ends up being used in lower-grade PET applications that are seldom recycled, breaking the circularity of this process.

In this context, the discovery of PET hydrolases^5,6^ has emerged as a promising and environmentally friendly approach for plastic biorecycling.^7,8^ These enzymes depolymerize PET into its main constituent monomers, which can then be used to resynthesize virgin PET and thus enable a fully circular plastic recycling process.^9^ Most PET hydrolytic enzymes are thermophilic,^5^ with optimal activities (∼65 °C) near the glass-transition temperature of PET (∼75 °C^10^), where the polymer chains become more flexible and are prone to enzymatic hydrolysis, due to the inability of PET hydrolases to degrade highly crystalline PET. However, a PET hydrolase from the mesophilic bacterium *Ideonella sakaiensis* 201-F6 (*Is*PETase) was described to degrade amorphous and semicrystalline PET with high efficiency at 30–35 °C.^11^

**Figure 1:**
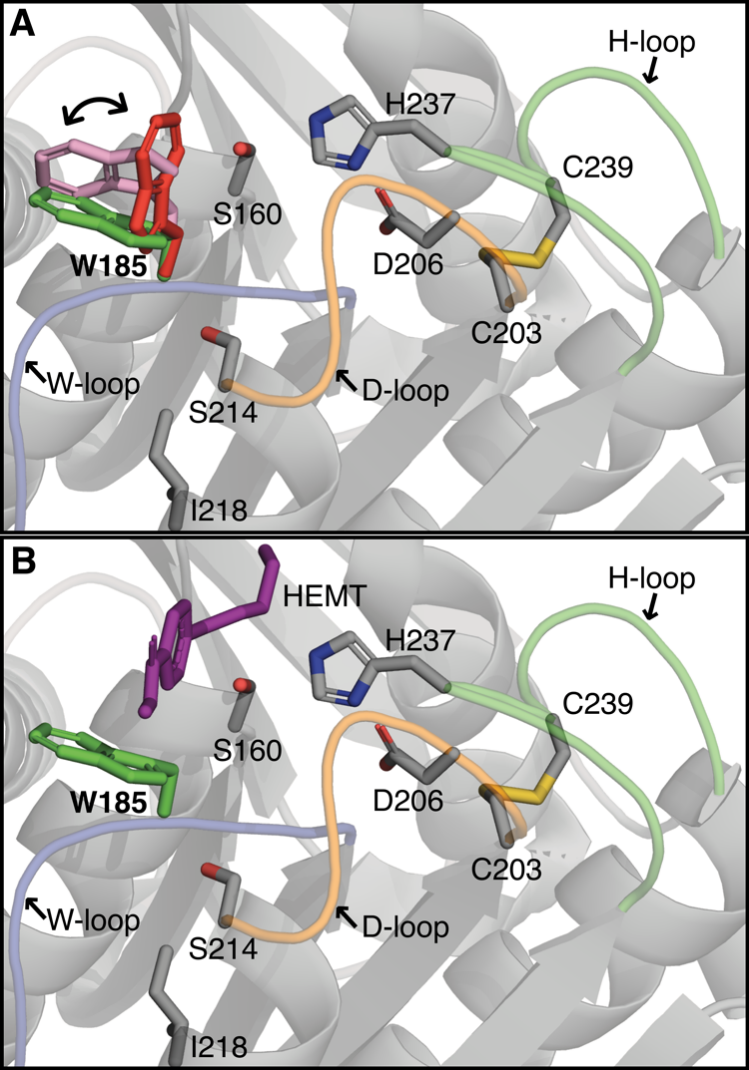
Multiple conformations of W185 observed in crystal structures of free and substrate-bound *Is*PETase. Cartoon representation of the active site of *Is*PETase (A) in the absence (PDB ID: 5XG0(14)) and (B) in the presence of the substrate analogue HEMT (PDB ID: 5XH3(14)). The key catalytic residues (S160, D206, H237), the active site disulfide bond (C203-C239), the W185 side chain, and the critical S214/I218 residues are shown in sticks. The different conformers of W185 are shown in red (conformer A), green (conformer B), and pink (conformer C). Relevant loops harboring W185 (W-loop), D206 (D-loop), and H237 (H-loop) are colored blue, orange, and green, respectively. The substrate analogue 1-(2-hydroxyethyl) 4-methyl terephthalate (HEMT) forms a T-shaped interaction with W185. Loops in cartoon representation were smoothed for visualization purposes.

While most known PET hydrolytic enzymes have been subjected to protein engineering via rational design or directed evolution, with substantial improvements in thermal stability and depolymerase activity,^12^ finding a “one size fits all” solution to improve the catalytic efficiency of any PET hydrolase has been elusive. A recent work^13^ presented a general mechanism for improving their catalytic activity based on the experimentally observed dihedral torsions of a conformationally dynamic tryptophan side chain in *Is*PETase, W185 (Figure 1), that is highly conserved in PET hydrolases in a loop within the active site (hereafter referred to as the W-loop). On a nearby helix, there are two residues, S214 and I218, which are unique to *Is*PETase, and in all other known PET hydrolytic enzymes correspond to histidine and phenylalanine.

Crystal structures of *Is*PETase in the absence of substrates (PDB ID: 5XG0(14)) show that the W185 side chain can adopt three conformations, termed A, B, and C, as illustrated in Figure 1, a phenomenon that has been described in the literature as “wobbling”.^15^ Substrate analogue-bound enzyme complexes (PDB IDs: 5XH2(14) and 5XH3(14)) show that this residue adopts conformer B for substrate binding, where it can form T-shaped, stacked, or parallel displaced interactions with one of the terephthalic rings of the substrate. Comparison of the crystal structure of *Is*PETase against a thermophilic PET hydrolase from a leaf-branch compost metagenome (LCC) suggests that the small size of S214 yields sufficient space for W185 to rotate to accommodate the substrate.^15^ Altogether, it is thought that tryptophan motion is relevant for substrate binding and product release. Consistently with this, the S214H mutant of *Is*PETase shows decreased activity compared to the wild-type enzyme.^14^

We note that there have been multiple elegant computational studies that have helped reveal the mechanisms of PET-degrading enzymes,^16−19^ more global conformational changes involved in catalysis,^20,21^ and contributed to associated engineering effort.^22−25^ However, corresponding studies of the conformational dynamics of this catalytically important tryptophan remain limited. Therefore, to explore the importance of the S214/I218 substitution for PET hydrolytic activity, doubly substituted variants of the strictly conserved histidine and phenylalanine residues in several PET hydrolases, including the mesophilic *Bur*PL from a *Burkholderiales* bacterium (H344/F348),^13^ and the thermophilic *Tf*Cut from *Thermobifida fusca* (H224/F228)^26^ and LCC (H218/F222),^27^ were generated based on the S214/I218 residues unique to *Is*PETase. All engineered variants have experimentally shown an increase in PET-degrading activity.^13^ Conversely, an H214/F218 variant of *Is*PETase exhibits a decrease in PET hydrolase activity.^13^ The enhancement in PET hydrolysis caused by the S214/I218 substitution is accompanied by a decrease in the optimal temperature for catalytic activity (*T*_opt_), suggesting that the substitutions impact either the local or global stability of these enzymes.

In this work, we employ molecular dynamics (MD) and well-tempered metadynamics^28^ simulations to explore the structural basis of the improved catalytic activity of *Bur*PL, *Tf*Cut, and LCC upon incorporation of the *Is*PETase-based S214/I218 double substitution. Our results show that these substitutions increase the flexibility of both active site loop regions harboring key catalytic residues, and the conformational plasticity of the conserved tryptophan within the W-loop. Notably, while all of the experimentally determined tryptophan conformers are accessible for all enzymes, the *Is*PETase-based S214/I218 substitution appears to facilitate population shifts to and from conformer A, as well as other conformations, such as a 180° side chain rotation that has not been observed in any solved crystal structures of PET hydrolases. Overall, our simulations provide evidence for a dynamical origin for the enhanced catalytic activity of the doubly substituted variants, affecting both loop dynamics and the conformational plasticity of the tryptophan.

## Methodology

### Preparation of Initial Structures

Initial coordinates for wild-type *Bur*PL (H344/F348), LCC (H218/F222), and *Tf*Cut (H224/F228) were obtained from the crystal structures deposited in the Protein Data Bank^29^ under PDB IDs: 7CWQ,^13^4EB0,^27^ and 5ZOA,^30^ respectively. For *Is*PETase, the initial structure corresponded to the recently solved structure of the single-point mutant S214H, PDB ID: 7CY0,^13^ where the serine residue in S214/I218 was replaced by the histidine that is strictly conserved in other PET hydrolytic enzymes. The doubly substituted versions of *Bur*PL (S344/I348), *Tf*Cut (S224/I228), and LCC (S218/I222), as well as the S214/I218 and H214/F218 variants of *Is*PETase, were generated using the Mutagenesis tool in PyMOL v. 2.4.2.^31^

Both *Is*PETase and *Bur*PL harbor an active site (C203-C239 for *Is*PETase, C333-C370 for *Bur*PL) and a C-terminal (C273-C289 for *Is*PETase, C404-C424 for *Bur*PL) disulfide bond. For *Tf*Cut and LCC, only the C-terminal disulfide bond (C281-C299 for *Tf*Cut, C275-C292 for LCC) is present. These disulfide bonds, which are present in the available crystal structures, were kept in all of the wild-type enzymes and doubly substituted variants.

Residue numbers for each enzyme are presented throughout the main text according to the full-length amino acid sequences of these proteins in the Uniprot database,^32^ with accession IDs: A0A0K8P6T7 (*Is*PETase), A0A1F4JXW8 (*Bur*PL), Q6A0I4 (*Tf*Cut), and G9BY57 (LCC).

### Molecular Dynamics Simulations

MD simulations were performed using the pmemd.cuda implementation of the AMBER 2020 simulation package,^33^ along with the ff14SB force field^34^ and the TIP3P^35^ water model for the parameters of protein atoms and water molecules, respectively. For each of the eight simulation systems, the protonation state of each residue at pH 9.0 was estimated with the H++ server,^36^ to match the experimental conditions in enzymatic degradation assays using either PET films or granules.^13^ The consistency of the estimated protonation states for the wild-type and doubly substituted variant of each enzyme was manually checked. The residues affected by the choice of pH include the catalytic histidine in all four enzymes (which would be protonated at pH 7), alongside E334 and H408 in *Bur*PL, which are not part of the active site cleft. The p*K*_1/2_ of these residues, employed by the H++ server to compute whether they should be protonated or not depending on the pH, is reported in Table S1. Then, all systems were solvated in truncated octahedral water boxes with 15 Å of padding and neutralized with Na^+^ or Cl^–^ counterions. A standard minimization, heating, and 10 ns equilibration protocol were performed, followed by NPT production at 1 bar and the experimentally determined optimal temperature of catalytic activity for each system^13^ (*i.e*. 303 K for *Is*PETase, 308 K for *Bur*PL, 323 K for LCC, and 333 K for *Tf*Cut). Constant temperature and pressure conditions for each system were controlled using a Langevin thermostat^37^ with a collision frequency of 1 ps^–1^, and a Berendsen barostat^38^ with a pressure relaxation time of 1 ps. MD simulations were run using a time step of 2 fs along with the SHAKE^39^ algorithm to constrain hydrogen-containing bonds, a 12 Å cutoff for nonbonded interactions, and the particle-mesh Ewald^40^ method for long-range electrostatics. Five 500 ns long replicas were produced for each of the wild-type and doubly substituted systems, for a total of 20 μs of MD simulations. The convergence of the simulations is shown in Figure S1.

### Well-Tempered Metadynamics Simulations

To study the torsional free energy associated with rotation of the conserved W-loop tryptophan, we performed eight 400-ns-long well-tempered metadynamics^28^ simulations (one per system), using the AMBER 2020^33^ simulation package and Plumed 2.7.^41^ We used as collective variables (CVs) the χ_1_ (N-C_α_-C_β_-C_γ_) and χ_2_ (C_α_-C_β_-C_γ_-C_δ2_) dihedral angles, which describe the torsional space of the tryptophan side chain. Metadynamics runs were started after 50 ns of NPT equilibration (following the same protocol and simulation settings as the MD). The backbone root-mean-square deviation (RMSD) of the helices and β-sheets of the protein structure, and the convergence of our metadynamics runs, are shown in Figures S2–S4. Simulations were performed using a bias factor of 12, a Gaussian width of 9° per CV, a Gaussian initial height of 0.1 kcal/mol, and a deposition rate of 1 ps^–1^. The convergence of these simulations was checked by monitoring the evolution of the CVs over time together with the free energy profile.

The free energy surface as a function of the two chosen CVs was calculated according to the theory of well-tempered metadynamics.^28,42^ Given the deposited bias potential, *V*
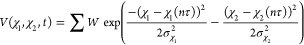
1where χ_1_ and χ_2_ denote the two selected CVs, τ denotes the time step, *n* denotes the number of steps until time *t* (so that the sum runs over all steps *n*τ < *t*), and σ_χ1_ and σ_χ2_ denote the Gaussian widths associated with the two CVs. In well-tempered metadynamics, *W* is also a function of time such that
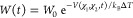
2Here, *W*_0_ is the initial height and Δ*T* is a parameter linked to the bias factor, γ, by the following equation
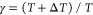
3Over long simulation timescales, this bias potential converges to the free energy, *F*
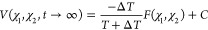
4with *C* being a constant that, for well-tempered metadynamics, vanishes for a long enough time (*i.e.*, once the diffusive state in the collective variable space has been reached). Finally, the free energy was evaluated as a function of the CVs using the Tiwary method^43^
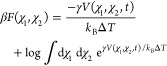
5

### Simulation Analysis

Simulation analysis was performed using the CPPTRAJ^44^ module from AMBER 2020^33^ and the Python library MDAnalysis.^45,46^ Snapshots were collected every 50 ps from the MD production runs for further analysis. Open cleft volume analysis was performed using POcket Volume MEasurer (POVME) 3.0,^47^ by considering 1000 frames over all replicas, taken every 2.5 ns of the production run. An initial sphere of radius 4 Å located at the center of mass between the α-carbon atoms of residues Y87 and M161 (*Is*PETase), Y217 and M291 (*BurPL)*, Y100 and S170 (*Tf*Cut), and Y95 and S165 (LCC) was used for the calculations. The residues forming the cleft are represented in Figure S5 and listed in Table S2. Root-mean-square fluctuations (RMSF) were calculated on the C_α_ atoms along the amino acid sequence of each protein relative to their averaged position over time. Hydrogen bonds for catalytic residues were calculated by considering a donor–acceptor distance cutoff of 3.5 Å and a donor–hydrogen–acceptor angle cutoff of 120°, considering all 25,000 frames collected per replica. The same number of frames was employed for calculating the change in distance between the C_γ_ atoms of the conserved tryptophan in the W-loop and the conserved tyrosine with which it forms an active site aromatic clamp.^20^ Finally, shortest path map (SPM) analysis was carried out by means of the *DynaComm.py* Python script,^48^ using the dynamic cross-correlation matrices and average inter-residue distance matrices for the MD simulations.

## Results and Discussion

**Figure 2:**
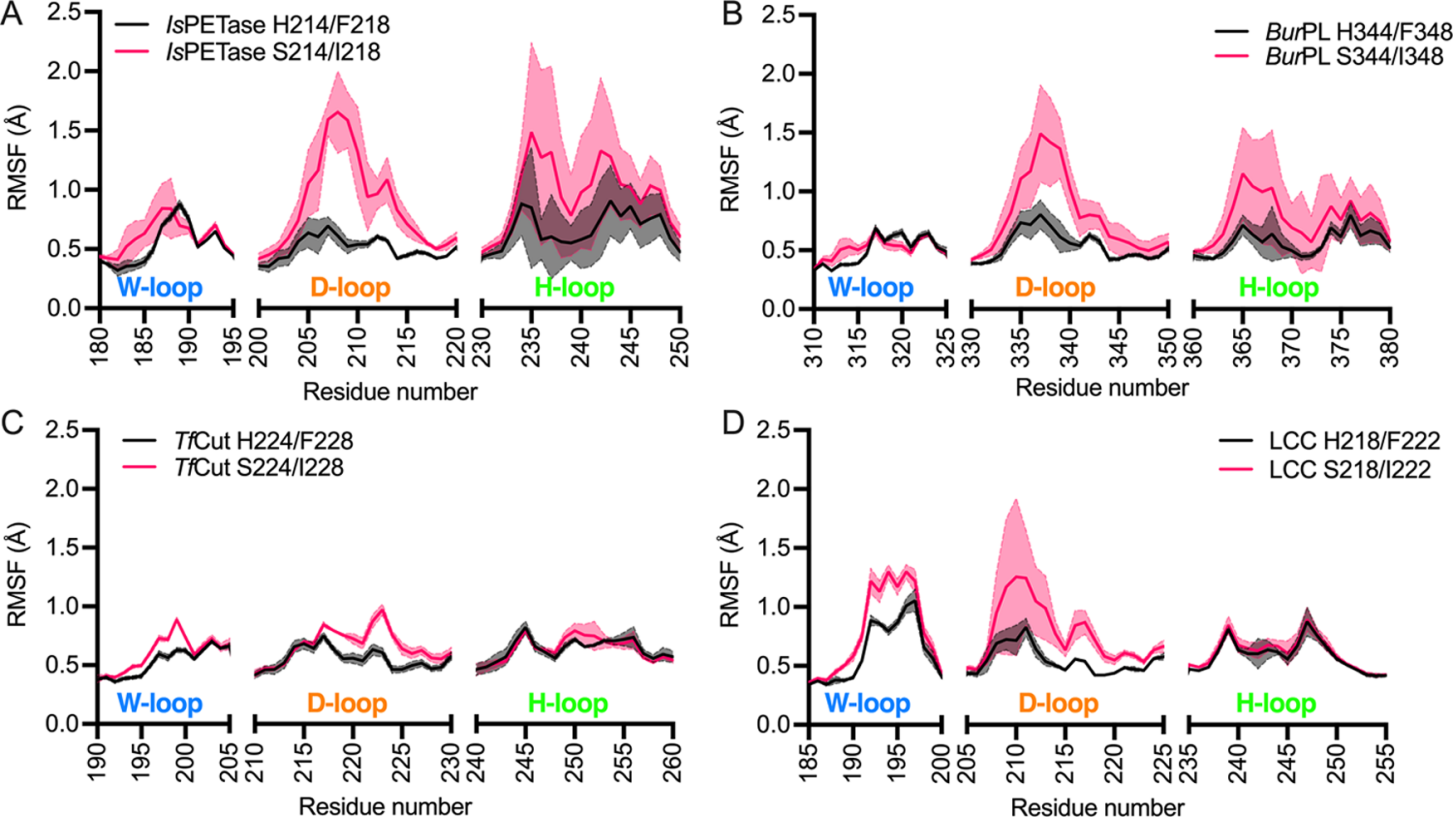
Effect of the *Is*PETase-based S214/I218 substitution on the local structural flexibility of PET hydrolases. (A–D) C_α_ atom root-mean-square fluctuations (RMSF, Å) of the flexible active site W-loop, D-loop, and H-loop (shown in Figure 1) during MD simulations of the *Is*PETase-based S214/I218 (pink) and H214/F218 (black) variants of (A) *Is*PETase, (B) *Bur*PL, (C) *Tf*Cut, and (D) LCC. The shaded area represents the standard deviation over 5 × 500 ns trajectories per system.

To determine the effects of the S214/I218 double substitution on PET hydrolase dynamics, we analyzed the root-mean-square fluctuations (RMSF) of the H214/F218 and S214/I218 variants of *Is*PETase and of the equivalent variants for *Bur*PL, *Tf*Cut, and LCC across a cumulative 2.5 μs of conventional MD simulations of each unliganded system (5 × 500 ns trajectories per system) at their respective temperature and pH optima. In all cases, comparison of the variants for each enzyme did not show significant differences in RMSF throughout the protein (Figure S6), except for the W- and D-loops (Figure 2), which harbor the conserved active site tryptophan (W185 in *Is*PETase) and the catalytic aspartic acid (D206 in *Is*PETase), respectively.

From the data shown in Figure 2, it is observed that in *Is*PETase, the substitution of S214/I218 by H214/F218 reduces the flexibility of the D-loop. Conversely, in the other PET-degrading enzymes, which harbor histidine and phenylalanine in their wild-type forms, the double substitution by serine and isoleucine increases the overall flexibility of the D-loop and, in the case of *Tf*Cut and LCC, the W-loop (although the precise magnitude of this effect varies by enzyme and specific loop). Moreover, the H-loop is more flexible in the serine and isoleucine variants of *Is*PETase and *BurPL* than in *Tf*Cut and LCC, despite harboring the active site disulfide bond connecting the D-loop and H-loop in the mesophilic enzymes (Figure 1). This could be explained by the longer extension of the H-loop in *Is*PETase and *Bur*PL than in the thermophilic enzymes, which enables the formation of a continuous cleft for substrate binding.^49^

**Figure 3:**
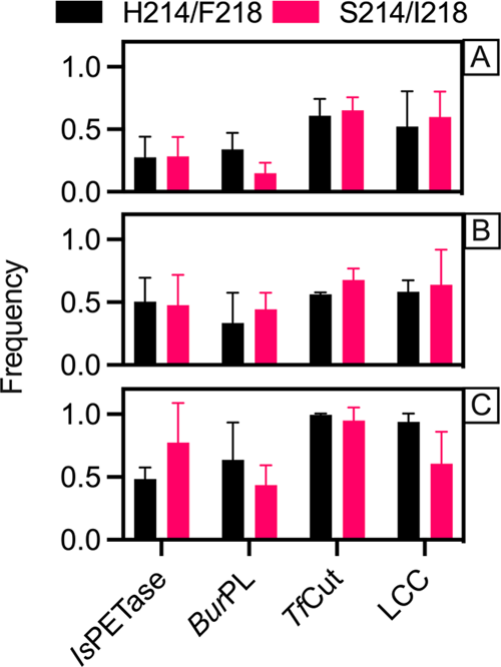
Frequency of interactions between key residues of the catalytic triad (Figure 1) during 5 × 500 ns simulations of each of the *Is*PETase-based S214/I218 and H214/F218 variants of PET hydrolases. Shown here are the relative frequencies of the interactions between (A) the hydroxyl group hydrogen of S160 and the N_ε_ of H237, (B, C) the N_δ_-bound hydrogen of H237 and each oxygen (B, O_δ1_; C, O_δ2_) from the carboxylic group of D206. The residue numbering corresponds to *Is*PETase. The error bars show the standard deviations across 5 × 500 ns conventional MD simulations of each system.

Despite the differences in loop flexibility of the key active site loops, two of which carry residues from the catalytic triad (Figure 1), we observe only subtle differences in the hydrogen-bonding patterns between the side chains of the catalytic serine (S160 in *Is*PETase) and histidine (H237 in *Is*PETase) and of the histidine and aspartate (D206 in *Is*PETase) for the *Is*PETase-based S214/I218 and H214/F218 variants of each system (Figures 3 and S7). It is worth noting that while the differences within each system are small, there is a more marked effect when comparing between different enzymes, and in particular between the mesophilic *Is*PETase and *Bur*PL and the thermophilic *Tf*Cut and LCC enzymes. Here, all mesophilic enzymes exhibit substantially lower occupancy of the key hydrogen-bonding interactions, and in particular the serine–histidine interaction (Figure 3A), than their thermophilic counterparts. This is consistent with the fact that the key active site loops in the mesophilic enzymes are overall more flexible than their thermophile counterparts (Figure 2), even after introducing the serine and isoleucine double substitution, and may account for the reduced catalytic activity of the mesophiles compared to their thermophilic counterparts.^12−14,20^

Following from this, we also calculated the volume of the active site cleft in all enzymes using POVME 3.0.^47^ The resulting averages and standard deviations are shown in Table 1. As can be seen from these data, with the exception of *Bur*PL, the *Is*PETase-based H214/F218 and S214/I218 variants of each system have similar active site volumes and fluctuations. However, there is quite a spread in active site volume and flexibility (using standard deviation as a proxy measure for flexibility), with *Bur*PL and *Tf*Cut having the most compact and least flexible clefts, and LCC having the largest and most flexible cleft. The average cleft volume depends on its shape, and on how many and how bulky the amino acid side chains which point inside the cleft are, whereas the volume fluctuations are intimately related to the structural flexibility of the cleft.

**Table 1:** Average Volumes of the Active Site Clefts of Each of *Is*PETase, *Bur*PL, *Tf*Cut, and LCC, during Our Simulations^a^

	open cleft volume (Å^3^)			
residues	*Is*PETase	*Bur*PL	*Tf*Cut	LCC
H214/F218	187 ± 93	165 ± 44	189 ± 58	235 ± 98
S214/I218	192 ± 70	228 ± 59	188 ± 59	230 ± 102

a All values were calculated using POVME 3.0^47^ and are presented as average values and standard deviations over 5 × 500 ns MD simulations per system, with snapshots taken every 2.5 ns of simulation time (2000 frames analyzed per system).

**Figure 4:**
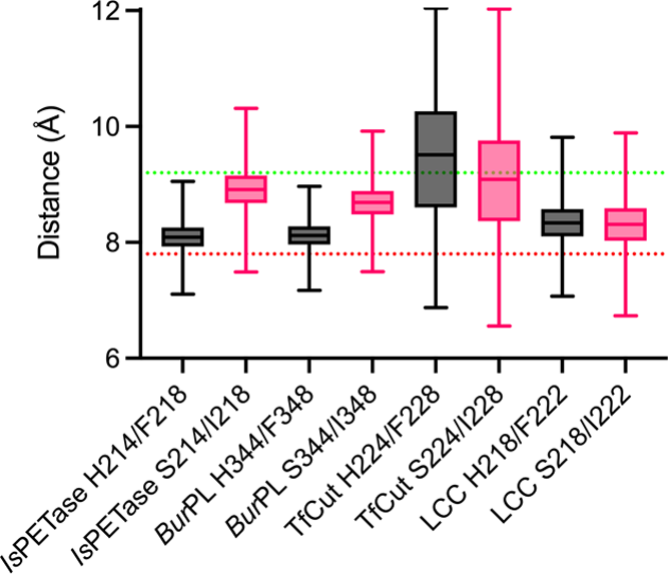
Box plot of the change in distance between the C_γ_ atom of the conserved tryptophan of the W-loop and the conserved tyrosine that completes the active site aromatic clamp, during 5 × 500 ns simulations of each of the *Is*PETase-based S214/I218 and H214/F218 PET hydrolase variants. The box length represents the interquartile range, which covers the central 50% of the data, and the horizontal line in the middle corresponds to the median, while the whiskers correspond to the minimum and maximum values. The dotted lines represent the experimental distances observed between Y87 and W185 in conformer B (red) and C (green) from *Is*PETase (PDB ID: 5XG0(14)).

For all enzymes, the conserved W-loop tryptophan is facing a tyrosine residue, forming an aromatic clamp into which one of the terephthalic rings of PET binds.^14,20^ When W185 in *Is*PETase (PDB ID: 5XG0(14)) adopts conformer B, the distance between the C_γ_ atoms of this residue and Y87 in the aromatic clamp is 9.2 Å, whereas for conformer C, the distance is 7.8 Å (Figure S8). Therefore, we analyzed the change in distance between these aromatic residues in all MD simulations. As shown in Figure 4, a median distance of ∼8.1 Å, similar to the experimental distance for the C conformer of W185, is obtained from the MD simulations for both *Is*PETase and *Bur*PL. In contrast, a median distance of 8.9 Å, resembling the experimental distance for conformer B, is observed for simulations of *Is*PETase and *Bur*PL with the S214/I218 substitutions. Finally, no differences are observed for the median distance of both variants of *Tf*Cut and LCC, with TfCut having a median distance 2 Å away from the crystallographic distance (PDB ID: 5ZOA(30)), whereas LCC is closer to the experimental distance (PDB ID: 4EB0(27)) (Figure S8).

**Figure 5:**
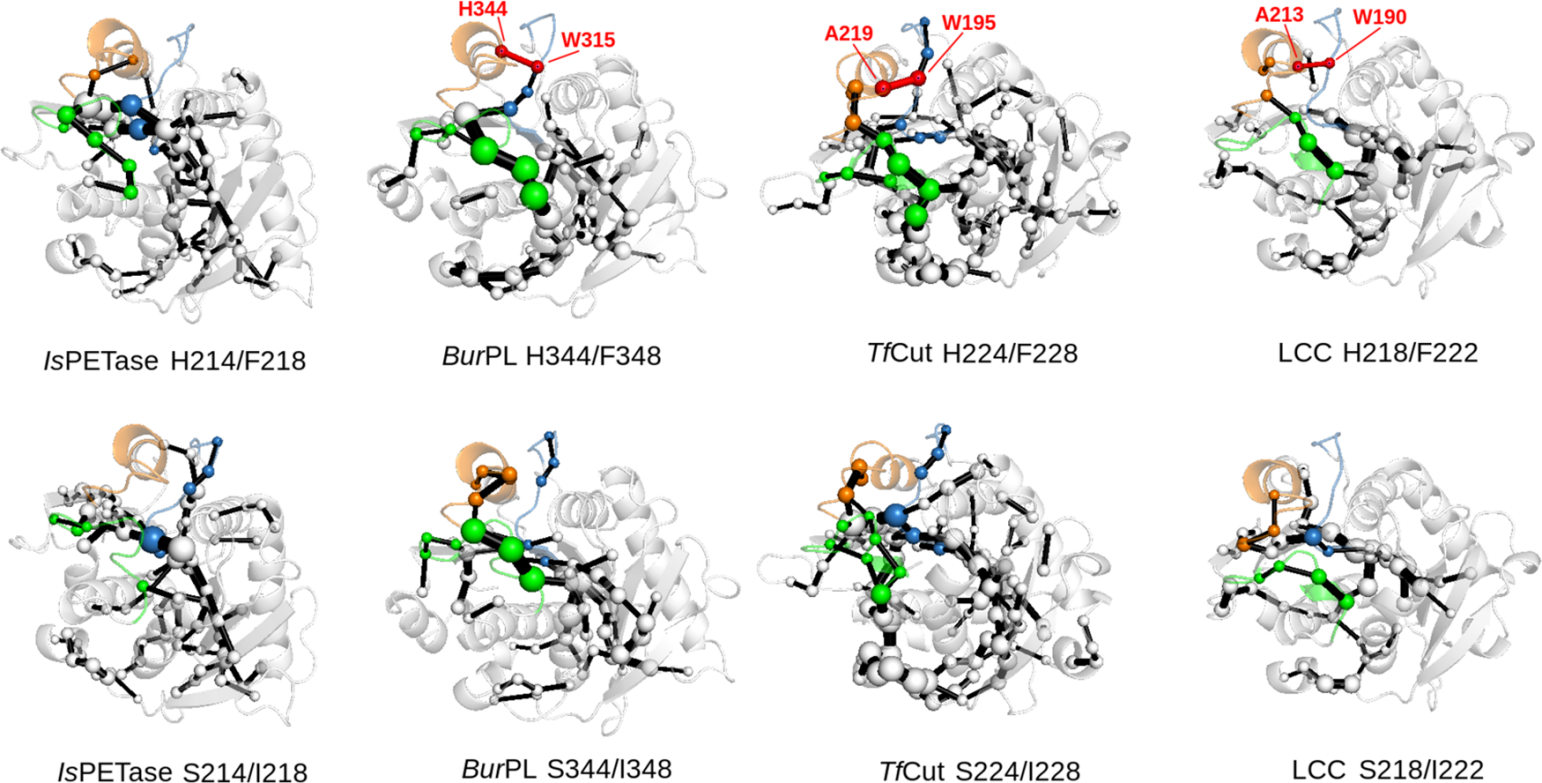
Representation of the shortest path map (SPM)^48^ highlighting allosteric communication pathways in the different PET hydrolases studied in this work. The SPM is represented by spheres (residues) and edges (connections between residues), with the size of the spheres and edges being proportional to the number of pathways involving a sphere or edge, and a larger sphere size effectively meaning more importance of that residue for allosteric communication. Loop D and the S214/I218-H214/F218 containing helix are represented in orange, loop H in green, and loop W in blue. The path connecting loops W and D is shown in red (with residues involved labeled).

Following from this, we performed shortest path map (SPM) analysis^48^ on the MD replicas to analyze the allosteric communication pathways that potentially connect the different active site loop regions. The SPM method allows for the calculation of the shortest pathways between all residues (the nodes) in the network. Nodes and edges (pathways between nodes) that are often used for communication between residues can then be identified, and these residues can therefore be considered as being important for regulating the global conformational dynamics of the enzyme. The calculated allosteric communication pathways are presented in Figure 5 and illustrate the differences between enzymes from different source organisms. Loop D and the mutation-hosting helix are only mildly important for the pathway of both variants of *Is*PETase. In *Bur*PL, loop H harbors significant residues whose motions are highly correlated. In *Tf*Cut, the network includes nodes on protein sections far from the active site.

When comparing the enzyme variants studied here, it is observed that the three loops, W, D, and H, appear linked to each other through different pathways on the identified networks. That is, with the exception of *Is*PETase, the SPM calculates a path connecting loops D and W in the cases of the histidine- and phenylalanine-containing variants (shown in red in Figure 5), whereas for *Is*PETase, this connection is absent in both H214/F218 and S214/I218 variants. For the H344/F348 variant of *Bur*PL, the connection takes place between the W-loop tryptophan (W315) and the H344/F348 histidine. In LCC and *Tf*Cut, the correlation takes place between the tryptophan (W190 and W195 respectively) in loop W, and A213 and A219, respectively. It is therefore possible that both the enhanced fluctuations of loops H and D, and the decreased correlations of the dynamics of the conserved tryptophan in loop W with residues on loop D may depend on the bulkier nature of histidine and phenylalanine, which may exert additional steric repulsion on loop W.

Next, we explored the effect of the *Is*PETase-based serine and isoleucine double substitution on the conformational stability of the side chain of the conserved tryptophan from loop W in our MD simulations. *Is*PETase residues S214 and I218, which correspond to histidine and phenylalanine in all other enzymes, are adjacent to W185 (Figure 1), with C_α_–C_α_ distances to this residue of ∼6 Å and ∼9 Å, respectively, based on PDB ID: 5XG0.^14^ Crystal structures of *Is*PETase (PDB ID: 5XG0(14)) have shown that W185 can rotate into conformers A (chain A, χ_1_ = 187.4°, χ_2_ = 20.7°), B (chain B, χ_1_ = 185.9°, χ_2_ = 79.3°), and C (chain C, χ_1_ = 188.2°, χ_2_ = 113.1°).^14^ Upon binding of substrate analogues, W185 adopts conformer B^14,15^ (Figure 1).

**Figure 6:**
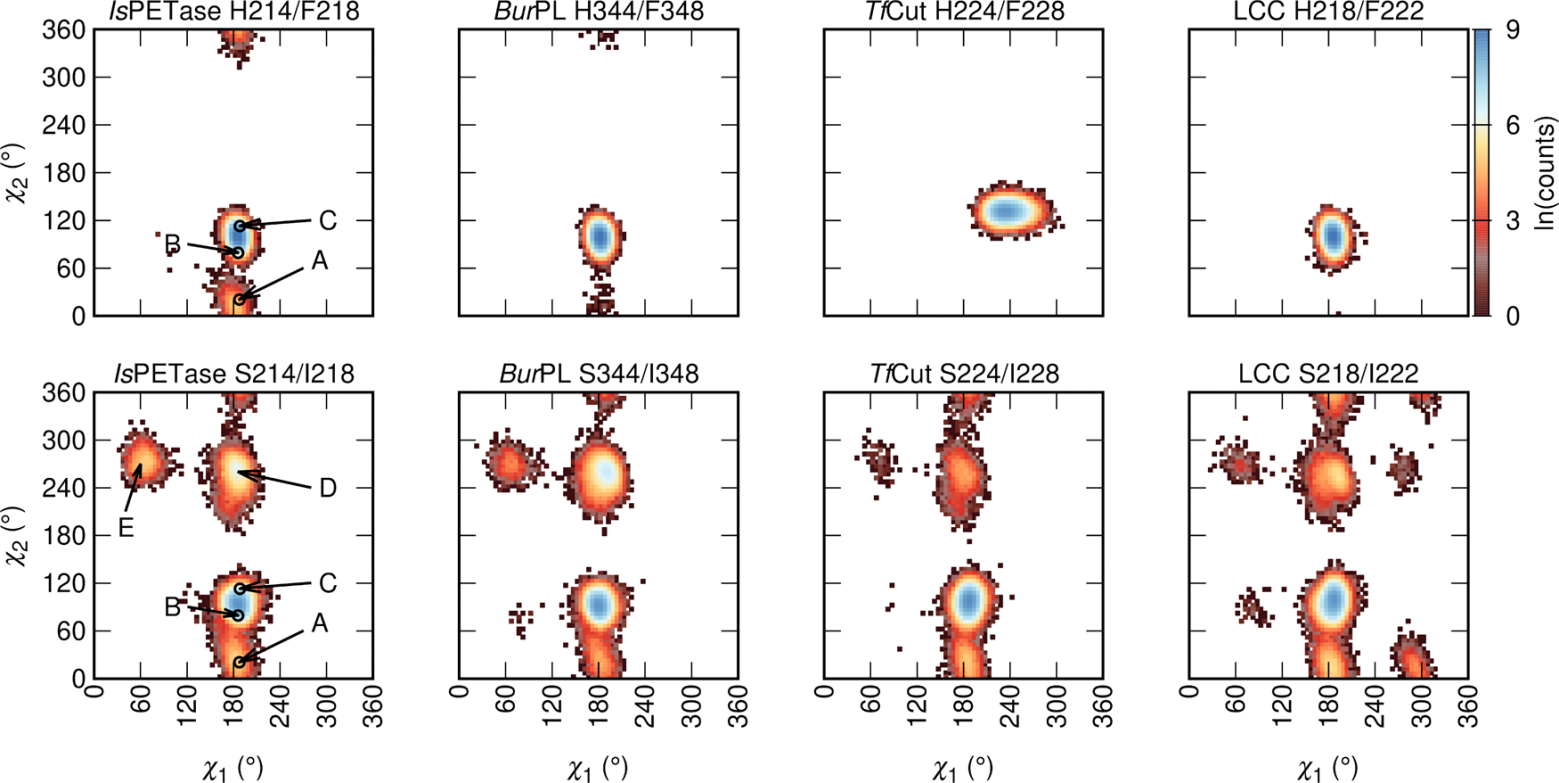
Torsional space explored by the W-loop tryptophan in the *Is*PETase-based H214/F218 and S214/I218 variants of PET hydrolases. The color gradient corresponds to the number of observations of a given χ_1_, χ_2_ conformer in all MD trajectories for each system, cumulatively totaling 2.5 μs simulation time per system (5 × 500 ns), with snapshots taken every 20 ps of simulation time. The arrows in *Is*PETase indicate the χ_1_, χ_2_ torsion angles of the A, B, and C conformers observed in the crystal structures of *Is*PETase, and the new D and E conformers observed in our simulations.

**Figure 7:**
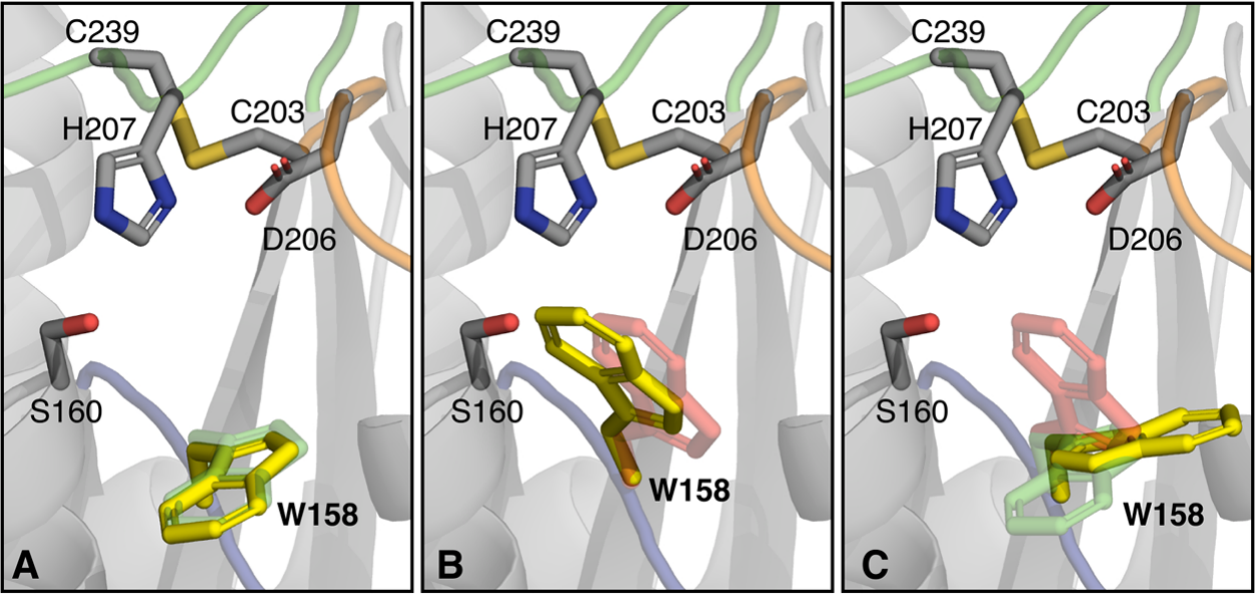
Representative structures of the different tryptophan conformations explored in our MD simulations. The residues of the catalytic triad and the W-loop tryptophan are shown in sticks, the MD-derived conformers for the W-loop tryptophan are shown in yellow, and the crystallographic conformers A and B are shown in red and green, respectively. (A, B) MD simulations exploring these conformers, whereas the conformer D from Figure 4, shown in (C), is only explored in the MD simulations and corresponds to a 180° rotation of the tryptophan side chain relative to conformer B.

To probe whether this plasticity is facilitated by the serine and isoleucine double substitution or by other features of the *Is*PETase scaffold, we calculated the χ_1_ and χ_2_ dihedral torsions of the corresponding tryptophan in all of the trajectories. From these data, it can be seen that the tryptophan side chain residue χ_1_ and χ_2_ dihedral angles are consistent with the B and C conformers of wild-type *Is*PETase in all of the H214/F218 and S214/I218 PET hydrolase variants (Figure 6). In contrast, the space created by the S214/I218 substitution and by the equivalent double substitution in *Bur*PL, *Tf*Cut, and LCC enables the tryptophan to rotate into the A conformer in all PET hydrolases, as well as explore new conformations (Figures 6 and 7) that have not been captured in experimental structures. This is consistent with the expected plasticity of this residue in the unliganded form of the enzyme.^14^ This plasticity is lost in the H214/F218 variant of *Is*PETase, consistent with the observed loss of activity of the *Is*PETase S187H variant.^14^ These data highlight that the S214/I218 residues alone are sufficient to facilitate W185 and the equivalent residue in all other enzymes to rotate, without the need for other modifications to the scaffold, and that this “wobbling effect” appears to be transferable to other PET hydrolases.

**Figure 8:**
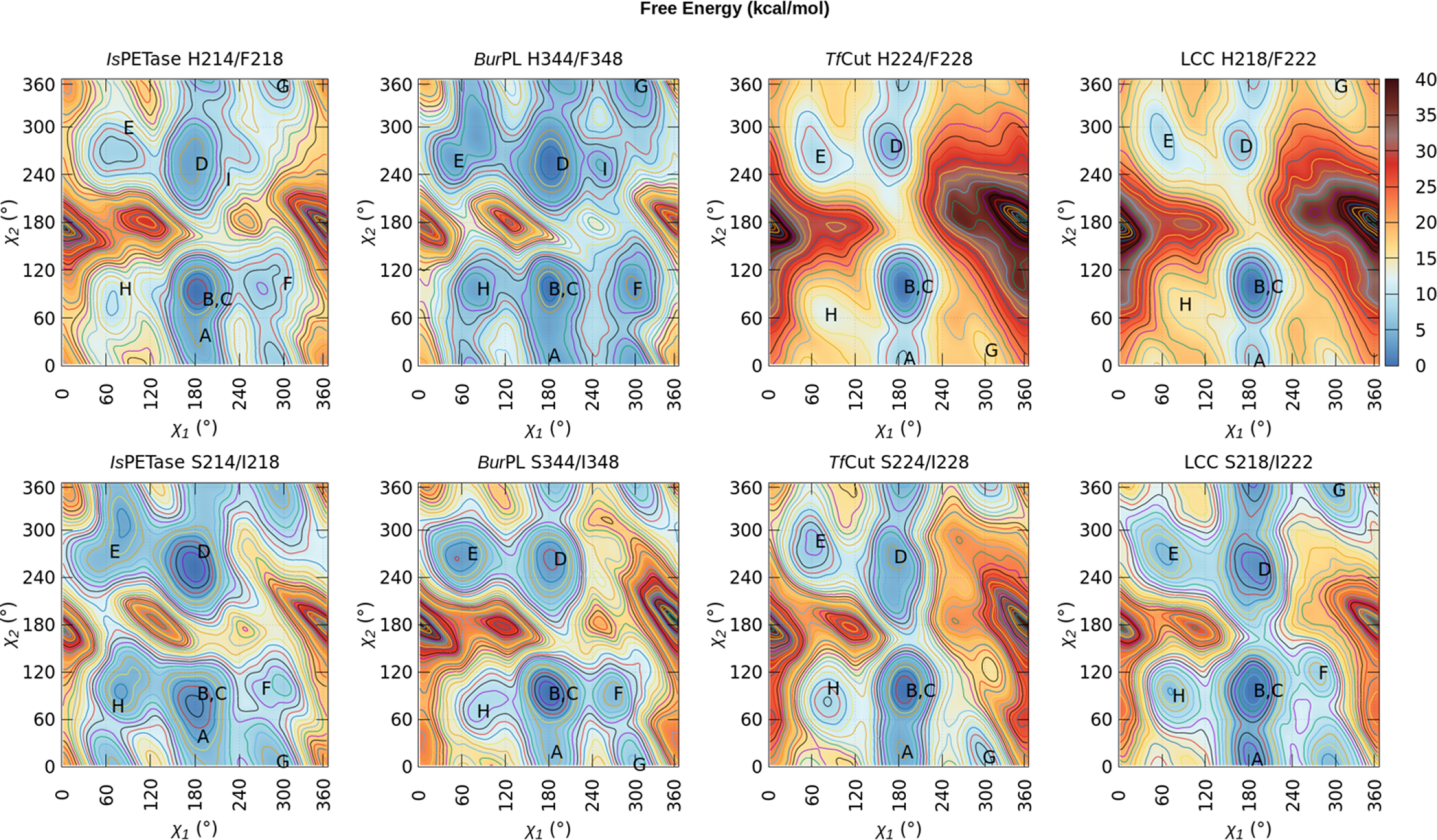
Free energy landscapes of the conformational space sampled by the side chain of the W-loop tryptophan in parallel-tempered metadynamics simulations of the *Is*PETase-based H214/F218 and S214/I218 variants of *Is*PETase, *Bur*PL, LCC, and *Tf*Cut. Free energy landscapes were generated as a function of the χ_1_ and χ_2_ dihedral angles. The minimum free energy value is set to zero in every graph. The corresponding energy barriers are tabulated in Table S1.

Given the observed conformational plasticity of the conserved tryptophan residue in loop W, we opted to fully explore its torsional space via well-tempered metadynamics simulations,^28^ in which the χ_1_ and χ_2_ dihedrals were used as collective variables (CVs). These simulations reached convergence within 400 ns, in terms of the diffusive behavior of the simulations (Figures S3 and S4). The free energy surfaces (FES) for each enzyme as a function of the two chosen CVs are presented in Figure 8. The overall landscape is conserved across all enzyme variants, with several common basins (labeled from A to I), separated by high energy barriers (Figure 8 and Table S3). The D, E, and F basins correspond to the tryptophan side chain being oriented toward S/H214 and I/F218. In the F basin, the tryptophan is also proximal to the aspartic acid, in the I basin to the histidine and in the G basin to the serine of the catalytic triad, respectively. The H218/F222 variant of LCC and *Tf*Cut (either as H224/F228 or S224/I228) feature neither the F nor the I basins. The I basin is only present in the H214/F218 variant of *Is*PETase and H344/F348 variant of *Bur*PL. For LCC and *Tf*Cut, the S218/I222 and S224/I228 mutations lower the barriers that connect the basins B, G, and E (Table S3). This is connected to an outward torsion of the tryptophan side chain (i.e., in the direction of the solvent), with the G basin corresponding to a conformation where the side chain shields the catalytic histidine from the solvent. The F basin in the S218/I222 mutant of LCC consists of the tryptophan side chain located in proximity to the catalytic residues.

Despite the overall topological similarities between the FES for each enzyme, important energetic differences are observed (Table S3). With the exception of *Bur*PL, the different FES suggest that conformers A and B in the serine and isoleucine doubly substituted variants are nearly isoenergetic, in comparison to the energetic favorability of the B conformer in the histidine and phenylalanine variants. Since the neighboring side chains for these conformers are almost the same for all organisms, this probably arises from subtle dynamical effects of the W-loop. Specifically, for LCC and *Tf*Cut, this change in the energetics of the FES is accompanied by reductions of 4.4 and 3.3 kcal/mol in the relative free energy difference between the B and A basins, respectively (Table S3). These results suggest that the *Is*PETase-based S214/I218 substitutions result in enhanced “wobbling” of the W-loop tryptophan in these thermophilic enzymes, consistent with their 2- and 3-fold increase in activity against PET films.^13^ Similarly, conformer D is energetically more favorable for the *Is*PETase-based S214/I218 PETase variants in comparison to the histidine and phenylalanine variants in almost all cases, except for *Bur*PL.

While our simulations are unable to elucidate the role of the new conformers observed by MD simulations for W185 in *Is*PETase and of the equivalent tryptophan residue in all variants of *Bur*PL, *Tf*Cut, and LCC, recent works have provided valuable insights. Plastic degradation assays and MD simulations by Guo et al.^50^ showed that PET polymers can bind in two different backbone conformations on the active site of *Is*PETase, *trans* (straight polymer conformation) and *gauche* (twisted polymer conformation), with a preferential selectivity for the *gauche* conformation that is abundant in PET films but absent in pretreated PET bottles. Interestingly, they design a *trans*-selective mutant (S238A) that has higher binding affinity to PET films than the wild-type enzyme and 3-fold higher activity against an all-*trans* PET substrate, which is enabled by W185 adopting conformer A to enable polymer binding in *trans*.^50^

In addition, a similar work on computational and experimental engineering of *Is*PETase to enhance its activity against PET^22^ shows that W185 in the wild-type enzyme adopts conformers different from B to establish parallel displaced interactions with the terephthalic ring in the lowest-energy, catalytically competent predicted pose. Conversely, the W159H/S238F mutant, with higher activity against PET, binds the substrate in a conformer similar to B to establish T-shaped interactions. Moreover, when modeling the binding of polyethylene-2,5-furandicarboxylate (PEF), an emerging bioplastic for replacing PET that is also degraded by *Is*PETase, W185 adopts conformers similar to D and A in the wild-type and W159H/S238F mutant enzymes, respectively.

## Conclusions

In this work, we have performed a combination of MD and well-tempered metadynamics^28^ simulations on four representative PET hydrolases with different optimal temperatures for hydrolytic activity, with the goal of providing an atomistic explanation on why the *Is*PETase-based S214/I218 substitution of two residues in the vicinity of the active site open cleft enhances the activity. Our investigation provides useful information regarding both the local dynamics of their active site. In particular, we have focused our attention on the flexibility of the active site and on the conformational plasticity of the side chain of a particularly important and highly conserved W-loop tryptophan, whose roles in the activity of these hydrolases have been previously demonstrated.^14,20^

Our MD simulations illustrate that the S214/I218 substitution results in enhanced flexibility of the active site loops (as demonstrated by their higher fluctuations), in particular loop D that is connected to the helix that harbors the substituted residues. In turn, this flexibility allows the W-loop tryptophan and the tyrosine residue with which it forms an active site aromatic clamp^20^ to occupy distances compatible with conformers for substrate binding in the two mesophilic enzymes studied. Even though the volume of the active site and its fluctuations over time are essentially unaltered by the mutations, we can speculate that the shape of the external part of the open cleft is, in the case of the *Is*PETase-based S214/I218 variants, amenable to accommodate the binding of a PET chain in the early stages of the overall degradation process. Validating this requires simulations that include the substrate docked into the binding pocket; whether this flexibility does in fact help substrate binding or product release will be a very interesting topic for future modeling studies. It is also clear that at the level of the global scaffold, the communication pathways differ by both organism and variant, which affects the flexibility of key loops and in turn the conformational sampling of the conserved tryptophan.

Regarding the conserved tryptophan, the unbiased MD simulations visited additional conformers A, D, and E for the *Is*PETase-based S214/I218 variants of PET hydrolases on a 500 ns timescale. Meanwhile, the free energy surfaces emerging from thorough exploration via metadynamics show how rotating through different basins is more likely in these PET hydrolase variants (which is true in general but it is particularly important for basins A and B), after elimination of the steric effects caused by the conserved histidine and phenylalanine residues.^14,15^ This enhanced rotation could be important for facilitating substrate binding and product release.

Prior work has proposed that these rotations of the conserved tryptophan are required to provide optimal aromatic interactions for the stabilization of substrate binding.^22^ Our insights complement these observations, suggesting that easy access and switch between different conformers of the conserved tryptophan is required for productive binding of different substrate conformations and possibly for product release to enable continued hydrolysis. The latter requires PET hydrolysis experiments in the presence of degradation products and simulations of enzyme–product complexes addressing the molecular mechanisms of exiting the active site of PET hydrolases.

Evolutionary conformational selection is not unique to PET hydrolases: *de novo* designed Kemp eliminases such as the HG3^51^ and KE07^52,53^ series also possess conformational flexible and catalytically important tryptophan side chains in their active sites, the conformations of which are optimized during directed evolution. Similarly, laboratory evolution of an organophosphate hydrolase, serum paraoxonase 1, showed similar conformational effects in key active site residues along the evolutionary trajectory.^54^ Similarly to these systems, our results suggest that engineering of PET hydrolases with mutations that enable conformational selection of the conserved tryptophan, such as S214/I218 and the S238A mutation in *Is*PETase, could lead to predefined active site conformations matching the polymer conformations in PET materials and enable higher catalytic efficiency. Further computational and experimental work is necessary to explore how different tryptophan conformations enable higher catalytic activity in natural and engineered PET hydrolases.

## Data Availability

The data underlying this study are available in the published article and its online supplementary material. A companion data package including input files, starting structures, and simulation snapshots has been submitted to Zenodo at the following DOI: 10.5281/zenodo.7158149.

## Supporting Information Available

RMSD of production runs for molecular dynamics and equilibration runs for metadynamics simulations; diffusive behavior of the metadynamics simulations; additional structural analysis; and free energy differences between the different conformational states (PDF)
