# Supplementary material for: Conformational Selection of a Tryptophan Side Chain Drives the Generalized Increase in Activity of PET Hydrolases through a Ser/Ile Double Mutation
Source: ACS Org Inorg Au. 2023 Jan 9;3(2):109–19. doi: 10.1021/acsorginorgau.2c00054 (PMC10080609; doi:10.1021/acsorginorgau.2c00054)
Supplement: Supplementary file 1 — gg2c00054_si_001.pdf [file gg2c00054_si_001.pdf]

Supporting Information for:

Conformational Selection of a Tryptophan Side Chain Drives the Generalized Increase in Activity of PET Hydrolases through a Ser/Ile Double Mutation

Alessandro Crnjar,[1,§] Aransa Griñen,[2,3,§] Shina C. L. Kamerlin,[1,4]* and César A. Ramírez-Sarmiento[2,3,]*

[1] Department of Chemistry–BMC, Uppsala University, Uppsala, BMC Box 576, S-751 23 Uppsala, Sweden. [2] Institute for Biological and Medical Engineering, Schools of Engineering, Medicine and Biological Sciences, Pontificia Universidad Católica de Chile, Av. Vicuña Mackenna 4860, Santiago 7820436, Chile. [3] ANID — Millennium Science Initiative Program — Millennium Institute for Integrative Biology (iBio), Av. Libertador Bernardo O'Higgins 340, Santiago 8331150, Chile. [4] School of Chemistry and Biochemistry, Georgia Institute of Technology, 901 Atlantic Drive NW, Atlanta, GA 30332-0400, USA.

[§] Both authors contributed equally to this work.

*Corresponding author email addresses: skamerlin3@gatech.edu and cesar.ramirez@uc.cl

Table of Contents

Supplementary Figures……………………………………………………………………S3

Supplementary Tables……………………………………………………………………S11

Supplementary References………………………………………………………………S14

Supplementary Figures

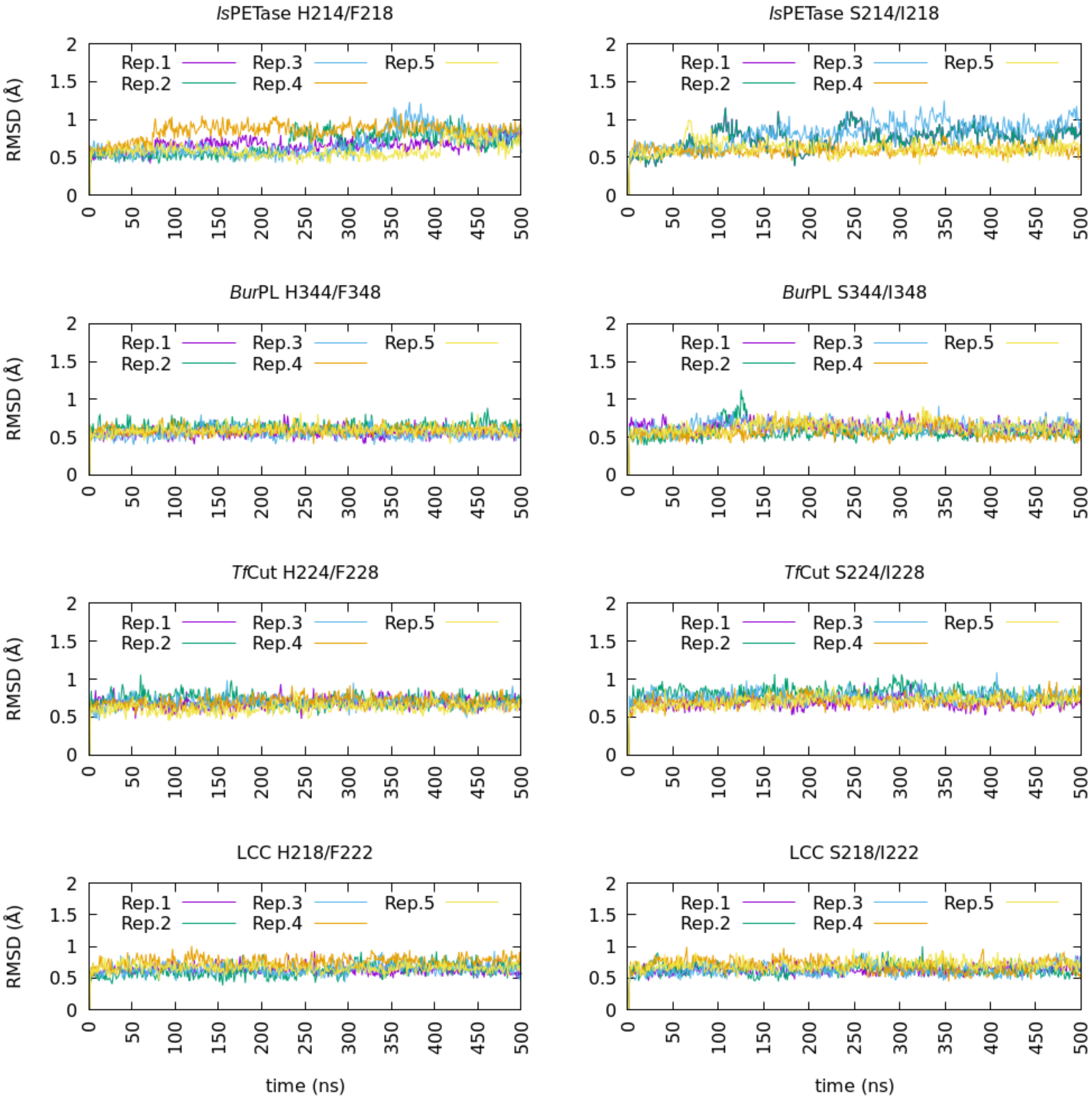

Figure S1. Helices and β-sheet backbone atom root mean square displacements (RMSD, Å), across 5 x 500 ns MD simulations of the *Is*PETase-based S214/I218 and H214/F218 variants (using *Is*PETase numbering) of *Is*PETase, *Bur*PL, *Tf*Cut and LCC.

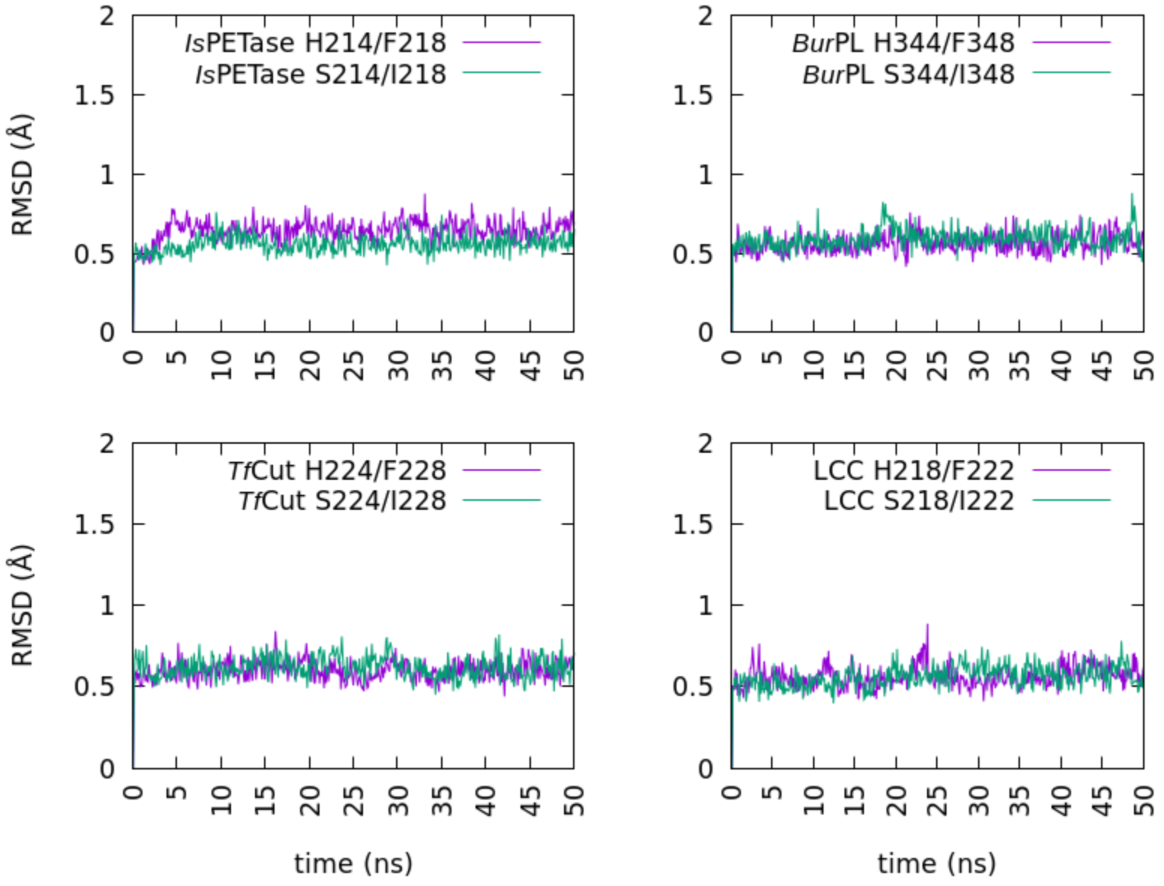

Figure S2. Helices and β-sheet backbone root mean square displacements (RMSD, Å) of the *Is*PETase-based S214/I218 and H214/F218 variants (using *Is*PETase numbering) of *Is*PETase, *Bur*PL, *Tf*Cut and LCC for the 50 ns long equilibration, ahead of the metadynamics simulations.

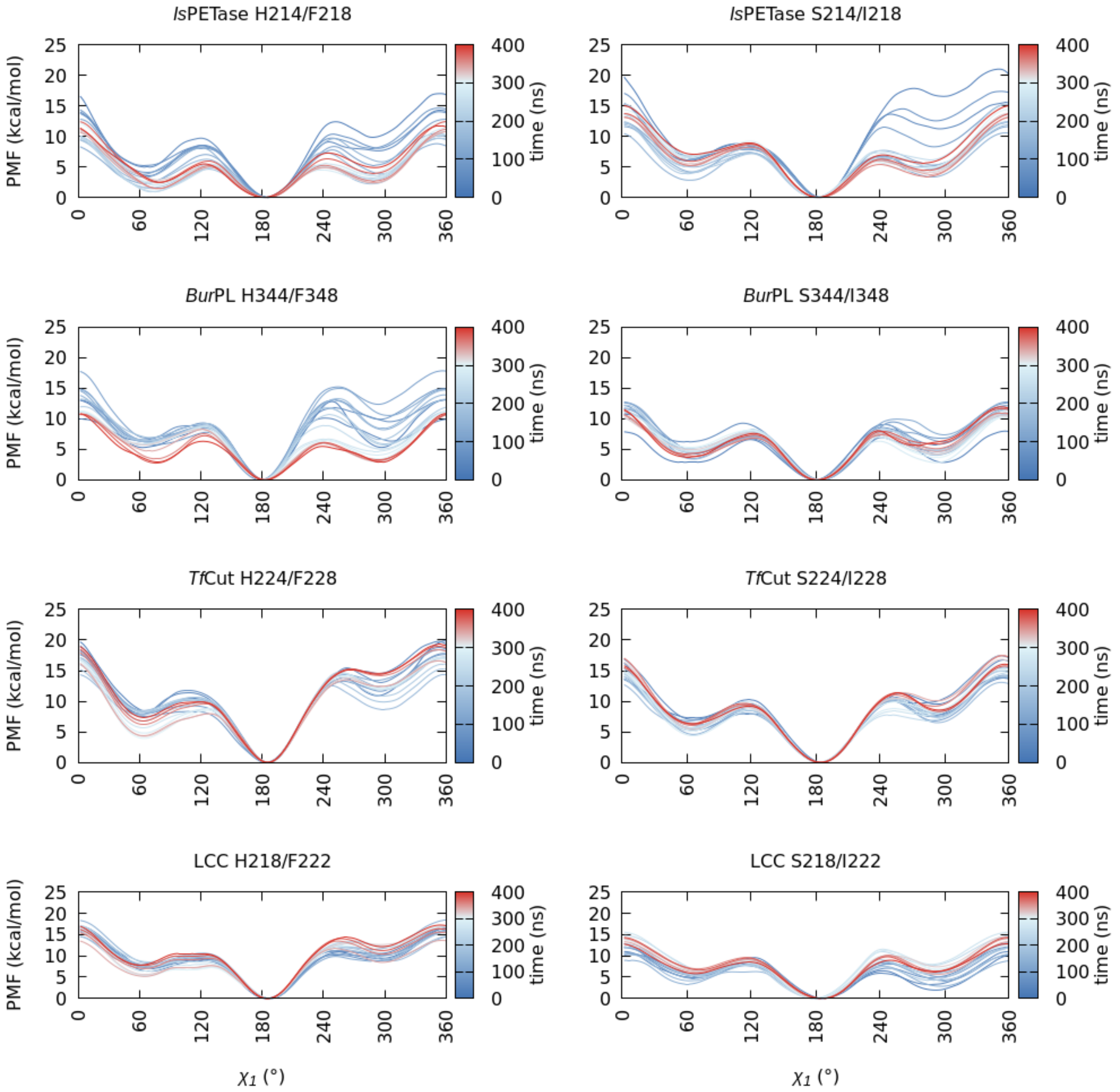

Figure S3. Evolution of Potential of Mean Force (PMF) of the eight metadynamics runs of the PET hydrolases, as a function of the collective variable χ_1 over the 400-ns-long metadynamics runs (the minimum of energy is set to 0 for all profiles). The profiles tend to stop changing after 350 ns, implying that convergence was reached within 400 ns, apart from a few unexplored regions in the CV space, which are not well-accessible for the side chain of the conserved tryptophan of the W loop due to steric repulsion with neighboring protein residues (see **Table S3** for the associated energy barriers).

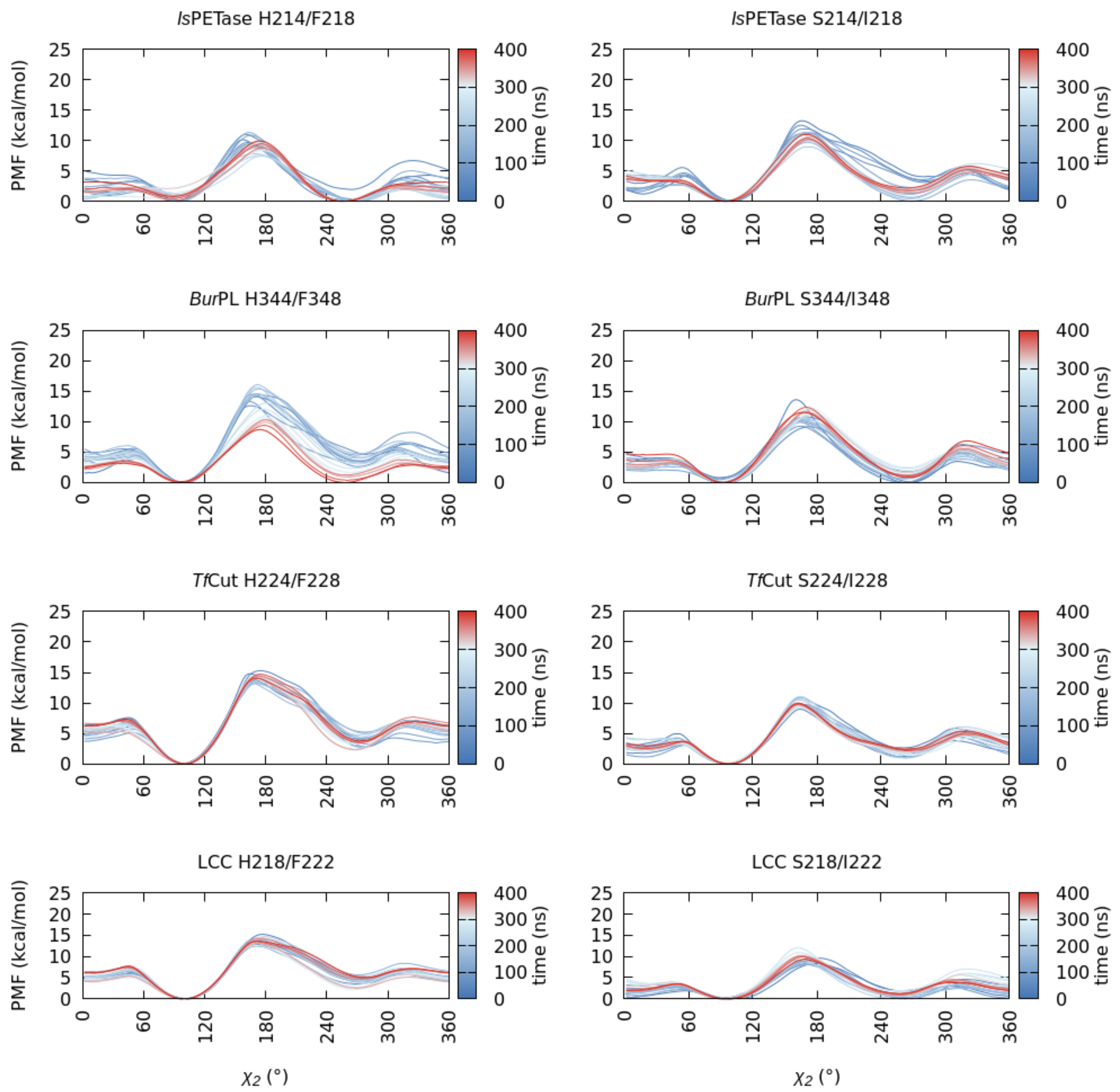

Figure S4. Evolution of Potential of Mean Force (PMF) of the eight metadynamics runs of the PET hydrolases, as a function of the collective variable χ_2 over the 400-ns-long metadynamics runs (the minimum of energy is set to 0 for all profiles). The profiles tend to stop changing after 350 ns, implying that convergence was reached within 400 ns, apart from a few unexplored regions in the CV space, which are not well-accessible for the side chain of the conserved tryptophan of the W loop due to steric repulsion with neighboring protein residues (see **Table S3** for the associated energy barriers).

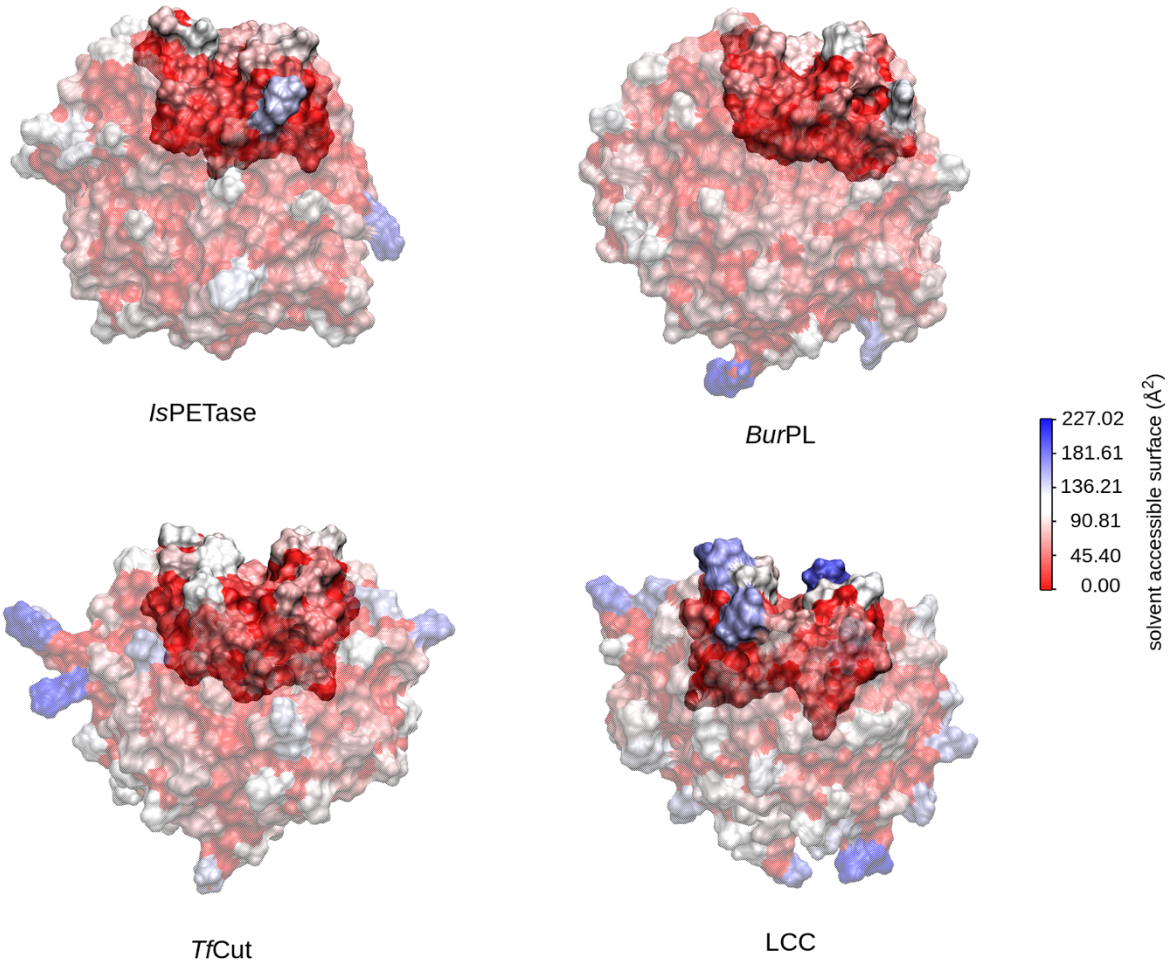

Figure S5. Solvent accessible surface of the cleft residues found with any atom at lower distance than 2.5 Å to the pseudoatoms used for the POVME[1] calculations of the active site volume (calculated with radius 1.4 Å and considering all but the backbone atoms). These residues are explicitly listed in **Table S2**. The solvent accessible surface of the rest of the protein is shown in transparent white.

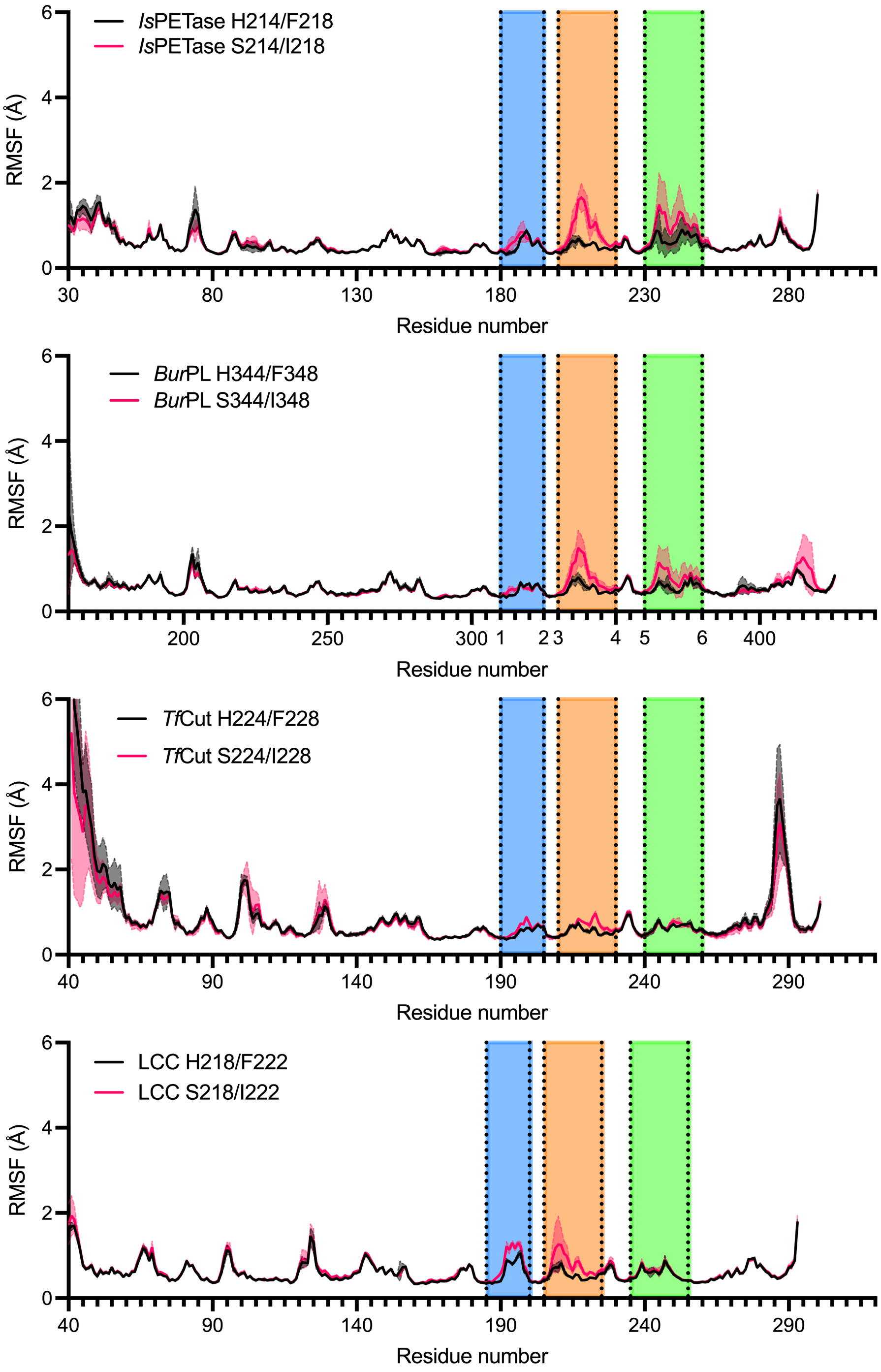

Figure S6. Full protein C_α-atom root mean square fluctuations (RMSF, Å) across 5 x 500 ns simulations of the *Is*PETase-based S214/I218 and H214/F218 variants (using *Is*PETase numbering) of *Is*PETase, *Bu*PL, *Tf*Cut and LCC. The shaded area represents the standard deviation across all replicas for each system. The blue, orange and green bars indicate the positions of the W-, D- and H-loops, respectively, using the same color coding as in **Figure 2**.

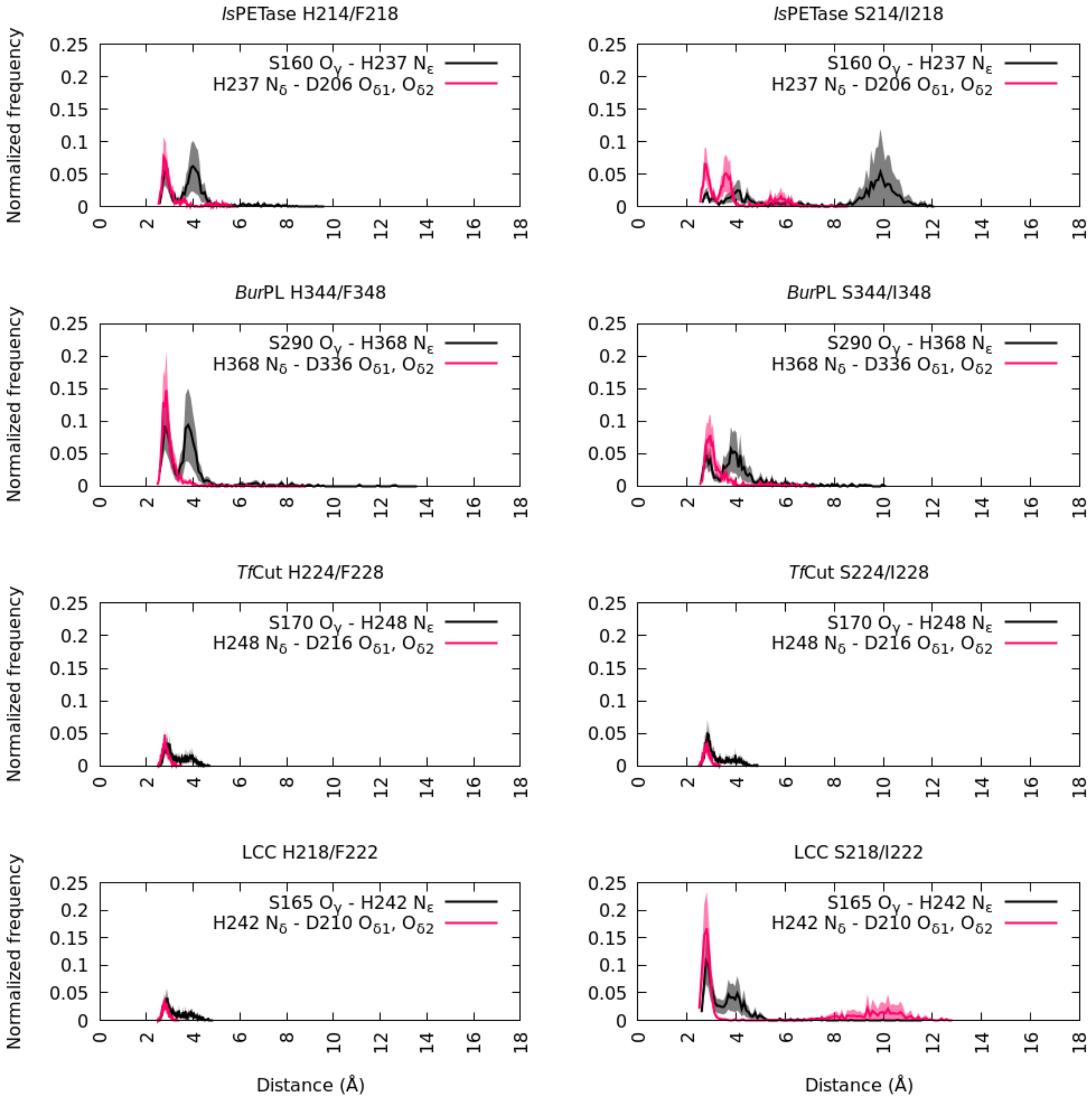

Figure S7. Distance distributions for the interactions between catalytic residues in PET hydrolases. Distance was measured for the hydroxyl group hydrogen of catalytic serine and the N_{ε} of catalytic histidine (black) and the N_{δ}-bound hydrogen of the catalytic histidine and the center of mass of the oxygens $O_{\delta 1}$ and $O_{\delta 2}$ from the carboxylic group of the catalytic aspartic acid (pink). Values of 8 Å and above correspond to frames in which the catalytic histidine is far away from the other two catalytic residues due to loop fluctuations. The shaded area represents the standard deviation across all replicas for each system.

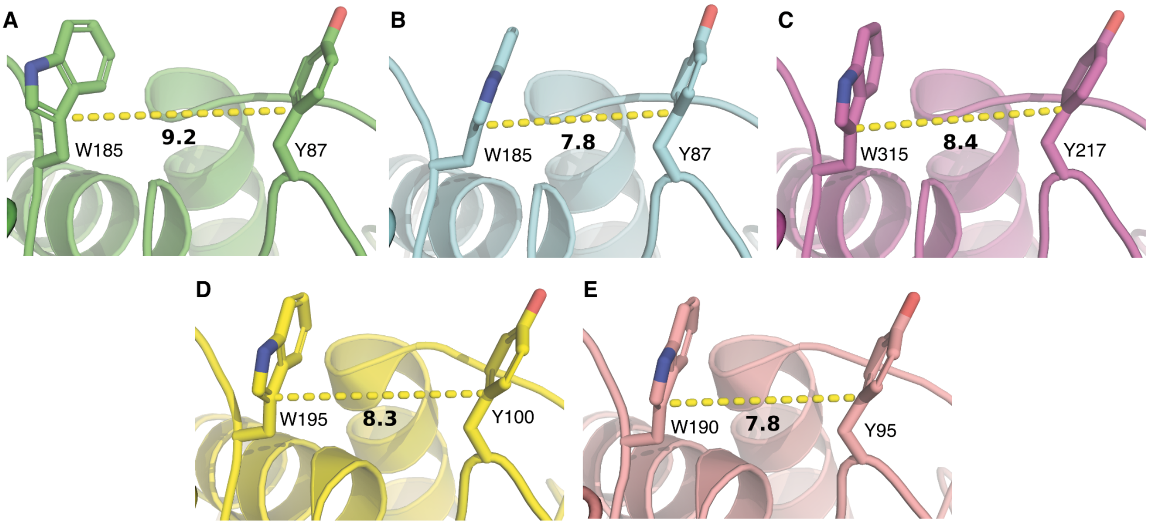

Figure S8. Crystallographic distances (in Å) between the C_γ-atom of the conserved tryptophan in loop W and of the tyrosine residue that constitutes the active site aromatic clamp of PET hydrolases. (A) *Is*PETase, W185 in conformer B (PDB ID: 5XG0[2]). (B) *Is*PETase, W185 in conformer C (PDB ID: 5XG0[2]). (C) *Bur*PL (PDB ID: 7CWQ[3]). (D) *Tf*Cut (PDB ID: 5ZOA[4]). (E) LCC (PDB ID: 4EB0[5]).

Supplementary Tables

Table S1. Residues affected by the choice of pH 9.0, with their p$K_{1/2}$ computed by the H++ server.[6]

**Enzyme**	**Key residues and associated pK$_{1/2}$**
*Is*PETase	H237 (7.4)
BurPL	E334 (8.7), H368 (7.8), H408 (7.4)
*Tf*Cut	H248 (8.5)
LCC	H242 (7.0)

Table S2. Cleft residues found with any atom at lower distance than 2.5 Å to the pseudoatoms used for the POVME[1] calculations of the active site volume.

***Is*PETase**	84, 85, 86, 87, 88, 89, 90, 93, 94, 113, 116, 117, 118, 119, 120, 121, 123, 124, 126, 158, 159, 160, 161, 164, 165, 168, 182, 183, 184, 185, 186, 187, 191, 206, 207, 208, 209, 214, 218, 237, 238, 240, 241
***Bur*PL**	194, 214, 215, 216, 217, 218, 219, 220, 223, 224, 241, 242, 243, 246, 247, 248, 249, 250, 251, 253, 256, 289, 290, 291, 292, 295, 312, 313, 315, 317, 338, 339, 340, 344, 368, 369, 372
***Tf*Cut**	96, 97, 98, 99, 100, 101, 102, 103, 104, 105, 106, 107, 109, 110, 124, 125, 126, 129, 130, 131, 132, 133, 136, 140, 167, 168, 169, 170, 171, 172, 175, 192, 193, 194, 195, 212, 213, 217, 218, 219, 220, 221, 224, 225, 247, 248, 249, 250, 251, 252, 253, 258
**LCC**	91, 92, 93, 94, 95, 96, 97, 98, 101, 102, 105, 118, 119, 121, 124, 125, 126, 127, 128, 131, 135, 163, 164, 165, 166, 167, 169, 170, 187, 188, 189, 190, 191, 207, 210, 211, 212, 213, 214, 215, 218, 219, 222, 241, 242, 243, 245, 246

Table S3. Relative free energy difference between different conformational states of the conserved tryptophan of the W loop during parallel-tempered metadynamics simulations of *Is*PETase, *Bur*PL, LCC and *Tf*Cut.[a]

**Basins**	***Is*PETase H/F**	***Is*PETase S/I**	***BurPL* H/F**	***BurPL* S/I**	**LCC H/F**	**LCC S/I**	***Tf*Cut H/F**	***Tf*Cut S/I**
**B/C, H**	9.7	1.7	3.3	8.4	15.1	6.0	12.4	8.8
**E, H**	2.2	0.1	0.3	5.1	6.1	0.4	4.2	2.3
**E, G**	0.3	1.2	0.6	3.5	3.8	0.2	6.8	1.1
**G, B/C**	7.2	3.0	3.6	6.8	12.8	5.4	15.0	7.6
**B/C, A**	3.8	1.8	4.2	5.3	7.4	2.5	7.7	3.8
**A, D**	0.3	3.7	4.2	2.6	1.1	0.5	2.9	0.2
**D, G**	3.7	5.0	3.6	4.1	6.4	3.4	10.2	4.1
**D, E**	4.0	3.7	3.0	0.6	2.6	3.6	3.5	3.0

[a]All energies are shown in kcal/mol. For simulation details, see the **Methodology** section.

Supplementary References

1. Wagner, J. R.; Sørensen, J.; Hensley, N.; Wong, C.; Zhu, C.; Perison, T.; Amaro, R. E., POVME 3.0: Software for Mapping Binding Pocket Flexibility. *J. Chem. Theory Comput.* **2017,** *13*, 4584-4592.

2. Han, X.; Liu, W.; Huang, J.-W.; Ma, J.; Zheng, Y.; Ko, T.-P.; Xu, L.; Cheng, Y.-S.; Chen, C.-C.; Guo, R.-T., Structural Insight into Catalytic Mechanism of PET Hydrolase. *Nat. Commun.* **2017,** *8*, 2106.

3. Chen, C.-C.; Han, X.; Li, X.; Jiang, P.; Niu, D.; Ma, L.; W., L.; Li, S.; Qu, Y.; Hu, H.; Min, J.; Yang, Y.; Zhang, L.; Zeng, W.; Huang, J.-W.; Dai, L.; Guo, R.-T., General Features to Enhance Enzymatic Activity of Poly(ethylene Terephthalate) Hydrolysis. *Nat. Catal.* **2021,** *4*, 425-430.

4. Dong, Q.; Yuan, S.; Wu, L.; Su, L.; Zhao, Q.; Wu, J.; Huang, W.; Zhuo, J., Structure-Guided Engineering of a *Thermobifida fusca* Cutinase for Enhanced Hydrolysis on Natural Polyester Substrate. *Bioresour. Bioprocess.* **2020,** *7*, 37.

5. Sulaiman, S.; Yamoto, S.; Kanaya, E.; Kim, J.-J.; Koga, Y.; Takano, K.; Kanaya, S., Isolation of a Novel Cutinase Homolog with Polyethylene Terephthalate-Degrading Activity from Leaf-Branch Compost Using a Metagenomic Approach. *Appl. Environm. Microbiol.* **2012,** *78*, 1556-1562.

6. Anandakrishnan, R.; Aguilar, B.; Onufriev, A. V., H++ 3.0: Atomatic p*K* Prediction and the Preparation of Biomolecular Structures for Atomistic Molecular Modeling and Simulations. *Nucleic Acids Res.* **2012,** *40*, W537-W541.
